# Sedentary behavior is associated with the mental health of university students during the Covid-19 pandemic, and not practicing physical activity accentuates its adverse effects: cross-sectional study

**DOI:** 10.1186/s12889-024-19345-5

**Published:** 2024-07-11

**Authors:** Bruna Carolina Rafael Barbosa, Luiz Antônio Alves de Menezes-Júnior, Waléria de Paula, Carolina Martins dos Santos Chagas, Elaine Leandro Machado, Eulilian Dias de Freitas, Clareci Silva Cardoso, Fernanda de Carvalho Vidigal, Luciana Neri Nobre, Luciana Saraiva da Silva, Adriana Lúcia Meireles

**Affiliations:** 1 Postgraduate Program in Health and Nutrition, Federal University of Ouro Preto, Ouro Preto, Brazil; 2 Postgraduate Program in Pharmaceutical Sciences, Federal University of Ouro Preto, Ouro Preto, Brazil; 3 Research and Study Group on Nutrition and Public Health, Federal University of Ouro Preto, Ouro Preto, Brazil; 4 Postgraduate Program in Nutrition and Health, Federal University of Lavras, Lavras, Brazil; 5 Department of Preventive and Social Medicine, Federal University of Minas Gerais, Belo Horizonte, Brazil; 6 Department of Medicine, Federal University of Juiz de Fora, Juiz de Fora, Brazil; 7 Department of Medicine, Federal University of São João del-Rei, São João del-Rei, Brazil; 8 Postgraduate Program in Nutrition and Longevity, Federal University of Alfenas, Alfenas, Brazil; 9 Postgraduate Program in Nutrition Sciences, Federal University of the Jequitinhonha and Mucuri Valleys, Diamantina, Brazil; 10 Postgraduate Program in Health Sciences, School of Medicine, Federal University of Uberlândia, Uberlândia, Brazil; 11 School of Nutrition, Federal University of Ouro Preto, Ouro Preto, Minas Gerais Brazil

**Keywords:** COVID-19, Anxiety, Depression, Sedentary behavior, Physical inactivity, Students

## Abstract

**Background:**

Movement behaviours, such as sedentary behavior (SB) and physical inactivity, have become a public health issue due to their implications for physical and mental health. The literature indicates that the university environment influences the movement behaviors of university students, and the strategies adopted during the pandemic may have favored a decrease in the practice of physical activity and an increase in the time dedicated to SB in this population. We aimed to evaluate the association of SB and moderate to vigorous leisure-time physical activity (MVPA) with presence of symptoms of mental disorders during the COVID-19 pandemic.

**Methods:**

This is a multicenter survey conducted with undergraduate students from eight Brazilian universities between October 2021 and February 2022 using an online questionnaire. The outcome variable was symptoms of anxiety and depression, assessed by the Depression, Anxiety, and Stress Scale-21. SB was assessed by total sitting time, being that individuals with ≥ 9 h/day were classified with high SB. The practice of MVPA was evaluated based on weekly frequency, duration, and type of exercise. Subsequently, the ratio between the time spent in MVPA (minutes/day) and the time spent in SB (hours/day) was calculated, being considered as cutoff point was the practice of 2.5 min of MVPA for each sedentary hour. To assess the association between the outcome and explanatory variables, multivariable logistic regression was performed.

**Results:**

A total of 8,650 students participated in the study, with an average age of 23.9 years (SD: ± 6.34). In the multivariate analysis, the odds of anxiety symptoms [OR: 1.37 (95% CI: 1.24–1.50)] and depression [OR: 1.61 (95% CI: 1.47–1.77)] were higher in individuals with ≥ 9 h of SB per day. In the analysis of the relationship between MVPA and SB, not engaging in 2.5 min of MVPA per hour of SB increases the odds of anxiety symptoms [OR: 1.44 (95% CI: 1.31–1.58)] and depression [OR: 1.74 (95% CI: 1.59–1.92)].

**Conclusion:**

The results suggest that SB is a risk factor associated with symptoms of anxiety and depression and that not engaging in MVPA exacerbates the negative effects of SB.

## Introduction

The COVID-19 pandemic has become a global public health problem, with social and economic repercussions for the population. As a result, the World Health Organization (WHO) advised governments to adopt social and health measures to reduce the spread of the virus [1–3]. In the area of education, there was the closure of higher education institutions and the inclusion of remote teaching, which impacted the routines and lifestyle of the university population [1, 4].

The sudden change in the university routine generated new demands that, together with the restrictions imposed in the initial phases of the COVID-19 pandemic, appear to have directly harmed the mental health of university students [5, 6]. Furthermore, it has been widely reported that during the pandemic there were unfavorable changes in the movement behaviors of university students, such as an increase in sedentary behavior (SB), characterized by low energy expenditure activities (≤ 1.5 metabolic equivalents - METs) [7] and a decrease in physical activity [3, 6, 8, 9].

Several studies have demonstrated that SB and physical inactivity, described as an activity level insufficient to meet physical activity recommendations [10], are associated with various physical and mental illnesses among university students, such as anxiety and depression [1, 8, 11], both mental disorders that have shown increasing trends in recent decades [12, 13]. There are some mechanisms that can explain the relationship between SB and the risk of depression, such as disturbances in biological pathways, which include central nervous system excitation [14]. Another explanation includes prolonged time in SB, for example watching television and using computers and smartphones, as this affects social interactions and causes withdrawal from interpersonal relationships and, therefore, increases the risk of depression and feelings of social anxiety [11, 14].

Some evidence suggests that physical activity may play a protective role against the negative effects of SB on all-cause mortality and mental health outcomes, with a low prevalence of stress and depression among university students [1, 3, 11]. In this context, it is important to investigate how physical activity can attenuate the effect of SB and mental health outcomes.

Although international research has sought to investigate the association between SB and mental health [1, 6], there is still a gap in knowledge regarding how this association manifests in Brazilian university students. There are no studies conducted in Brazil associating SB with the mental health of university students, especially during the COVID-19 pandemic. This gap is significant because available studies generally focus on other population groups, such as adolescents, adults, and the elderly, or do not consider mental health as an outcome variable [15]. Additionally, most existing research examines physical inactivity and SB in isolation rather than considering their combined effects on mental health. Our study seeks to fill this gap by examining the simultaneous impact of SB and physical activity on the symptoms of anxiety and depression of college students during the pandemic in Brazil. This dual focus is crucial, as physical activity can mitigate the negative effects of SB by promoting physical and mental well-being through the regulation of the hypothalamic-pituitary-adrenal (HPA) axis, neuroendocrine system responsible for managing stress in the body [16–18], as well as explaining the negative effects of SB on mental health.

Therefore, the innovative aspects of this study lie in its specific focus on the Brazilian university student population during the unique context of the COVID-19 pandemic and its comprehensive approach to evaluating both SB and physical activity in relation to mental health. By addressing these gaps, our research provides new insights that can inform targeted interventions and support strategies for this vulnerable group.

Considering that studies associating SB with mental health are incipient and still poorly explored in the specific context of university students, this study hypothesizes that university students with SB are more likely to present symptoms of anxiety and depression, and that not practicing in moderate to vigorous leisure-time physical activity (MVPA) may exacerbate the negative effects of SB.

## Methods

### Design, study population and data collection

This is a cross-sectional study with data from the multicenter survey “Symptoms of anxiety and depression disorder among university students in Minas Gerais: prevalence and associated factors,” also called Project on Anxiety and Depression in University Students (PADu-multicenter). The survey was carried out with students enrolled in the second semester of 2021 in face-to-face and distance learning undergraduate courses at eight public universities in Brazil. The PADu-multicenter project was approved by the Research Ethics Committee of the coordinating center through under protocol number 43027421.3.1001.5150, as well as by the Research Ethics Committees of all participating universities.

PADu-multicentric study is conducted by the Universidade Federal de Ouro Preto (UFOP) in partnership with the Universidade Federal de Minas Gerais (UFMG), Universidade Federal de Uberlândia (UFU), Universidade Federal de Juiz de Fora (UFJF), Universidade Federal de São João del-Rei (UFSJ), Universidade Federal de Lavras (UFLA), Universidade Federal dos Vales do Jequitinhonha e Mucuri (UFVJM), and Universidade Federal de Alfenas (UNIFAL-MG).

The eligible population of the PADu-multicenter study consisted of 118,828 students regularly enrolled in undergraduate courses offered by participating universities during any academic period. Among them, 7.3% responded to the questionnaire, a response rate consistent with the rates reported in the literature for similar studies. The final sample of the PADu-multicenter study comprised 8,650 students. Power calculations indicated that our study was adequately powered to identify meaningful differences and associations, ensuring the robustness and reliability of our findings (power above 90%).

Students aged 18 years or older of both genders were included, while those who did not complete the entire questionnaire, graduate students, residents, and students on leave from academic activities or on exchange during data collection were excluded. Recruitment involved sending emails containing informative invitations and questionnaire links. As a communication, awareness, and participant recruitment strategy, the research was widely publicized on the websites and social networks of the participating universities, as well as through tutoring programs, laboratories, study and research groups, centers, and academic directories.

Data collection lasted for three months at each university, conducted between October 2021 and February 2022, using a structured and self-administered questionnaire divided into thematic modules: sociodemographic and academic characteristics, lifestyle habits, and health conditions. Students who voluntarily agreed to participate in the study completed the questionnaire provided on Google Forms^®^. Participation in the study began at the moment of accessing the questionnaire, upon agreement via an online check with the Informed Consent Form, which was presented electronically and available for download. All questionnaire items were presented, detailing important points and addressing possible questions.

Further details about the methodology of the PADu-multicenter can be found in a previous publication [19].

### Study variables

#### Outcome variables: mental health

The variables “symptoms of anxiety” and “symptoms of depression”, considered outcomes, were assessed using of the Depression Anxiety Stress Scale-21 (DASS-21), translated and validated for the Portuguese language by Vignola and Tucci. The DASS-21 is a self-report instrument composed of three subscales, with seven questions each, which assess feelings presented by the individual in the last week [20]. Questions 3, 5, 10, 13, 16, 17 and 21 refer to the depression subscale; items 2, 4, 7, 9, 15, 19 and 20 form the anxiety subscale; and questions 1, 6, 8, 11, 12, 14, 18 make up the stress scale.

In the psychometric validation of the DASS-21 for the Brazilian context, strong correlations were observed, with a Cronbach’s alpha of 0.92 for the depression subscale, 0.90 for the stress subscale, and 0.86 for the anxiety subscale, indicating good internal consistency for each subscale of the DASS-21 [20]. The results of Vignola and Tucci (2014) support the reliability and validity of the Brazilian Portuguese version of the DASS-21, providing evidence that each subscale measures its intended to measure, in other words, the quality and ability to assess emotional states separately, ensuring the legitimacy of each subscale as an independent measure.

DASS-21 response scoring is based on a four-point Likert-type scale, ranging from 0 (did not apply at all) to 3 (applied a lot or most of the time). The final result of the scale is obtained through the sum of the scores of the items of each subscale multiplied by two to correspond to the score of the original scale (DASS-42), generating scores that allow the classification of symptoms into the levels “normal”, “mild”, “moderate”, “severe”, and “extremely severe”.

For the present study, only symptoms of anxiety and depression were evaluated and subsequently reclassified into absence “no” (normal and mild) and presence “yes” (moderate to extremely severe).

### Explanatory variables: movement behaviors

#### Sedentary behavior

SB was assessed by total sitting time through the questions “Currently, from Monday to Friday, how many on average (in hours) do you spend sitting (include the time used for cell phone, TV, computer, tablet, books, car, and bus) per day?” and “Currently, on weekends, how long (in hours) on average do you spend sitting (include time spent on your cell phone, TV, computer, tablet, books, car and bus) per day?” These questions were adapted from a similar question to the long version of the International Physical Activity Questionnaire (IPAQ) [21].

For analysis purposes, total sitting time was classified in two ways: every 3 h/day (< 3 h; 3–6 h; 6–9 h; ≥ 9 h) and from the cut-off point of ≥ 9 h/day (< 9 h and ≥ 9 h), this second classification is based on a meta-analysis involving over 1 million participants from 19 studies, suggesting that higher levels of SB are associated with increased risk of all-cause mortality in adults [22]. It is important to highlight that there are no specific recommended cutoffs for SB in adults concerning mental health outcomes. Although the cutoff by Ku et al. [22] is not specifically associated with mental health, we chose to use it due to its broad acceptance in the literature and its demonstrated consistency concerning other health outcomes, such as cardiovascular diseases and all-cause mortality.

#### Leisure-time physical activity

The practice of leisure-time physical activity was assessed based on weekly frequency, duration and type of exercise performed, considering the following questions: “What type of physical exercise or sport did you practice?”, “How many days a week do you usually practice this physical exercise or sport?” and “On the day you practice this exercise or sport, for how long (in minutes) does this activity last?”. The following were classified as moderate intensity practices: walking, treadmill walking, cycling, weight training, stretching, yoga, pilates, water aerobics, swimming, fights and martial arts (jiu-jitsu, karate, judo, boxing, muay thai, capoeira), cycling (includes ergometric), volleyball/footvolley, dance (ballet, ballroom dancing, belly dancing, forró and axé) and others. On the other hand, running, treadmill running, crossfit, functional, aerobic gymnastics (spinning, step, jump), football/futsal, basketball, tennis, HIT (high intensity interval training) and team sports were classified as vigorous intensity exercises [23, 24].

To determine the average daily practice variable of MVPA, the weekly frequency of MVPA (0 to 7 days) was multiplied by the daily MVPA time (in minutes), divided by 7. Subsequently, the ratio between the time spent in MVPA in the students’ leisure and SB time. For this, the time spent practicing MVPA was divided by the hours in SB according to the following equation: average daily time in MVPA (minutes/day)/time in SB (hours/day). Then, the variable was classified according to the recommendations of Chastin and collaborators [25], who suggest practicing at least 2.5 min of MVPA for every sedentary hour, as a way to reduce the impacts of SB on health and the risk of mortality.

### Covariates

Sociodemographic, academic, and health variables were used to describe the sample and to adjust the analysis of the association between SB and mental health, namely: biological sex (male and female), age (18 to 20 years, 21 to 22 years, 23 to 25 years and ≥ 26 years), race/skin color (white, brown, black and yellow/indigenous/other), sexual orientation (heterosexual, homosexual, bisexual and asexual/other), marital status (single, married/in a union stable and widowed/divorced), housing (with family members and without family members), education of the head of the family (no education or incomplete primary education, complete primary education or incomplete secondary education, complete secondary education or incomplete higher education, and complete higher education), and total family income (≤ 1 to 2 minimum wages, 3 to 5 minimum wages, 6 to 10 minimum wages and > 10 minimum wages). The variable evaluated in the academic domain referred to the course’s area of knowledge (life sciences, exact sciences, human and social and applied sciences). Nutritional status was assessed based on body mass index (BMI), obtained through anthropometric measurements of weight and height, self-reported by participants. The classification was carried out based on the values recommended by the WHO for adolescents [26], adults [27] and the elderly [28]. Individuals classified as underweight and eutrophic (BMI < 24.9 kg/m^2^) were grouped into the “not overweight” category and those classified as overweight and obese (BMI ≥ 25 kg/m^2^) into the “overweight” category. More details about the nutritional status classification methodology can be found in Barbosa and collaborators [29].

### Statistical analysis

For sample description and data comparison, sociodemographic variables, movement behaviors, and anthropometric profile were analyzed using frequency distribution and Pearson’s chi-square test, respectively.

**Fig. 1:**
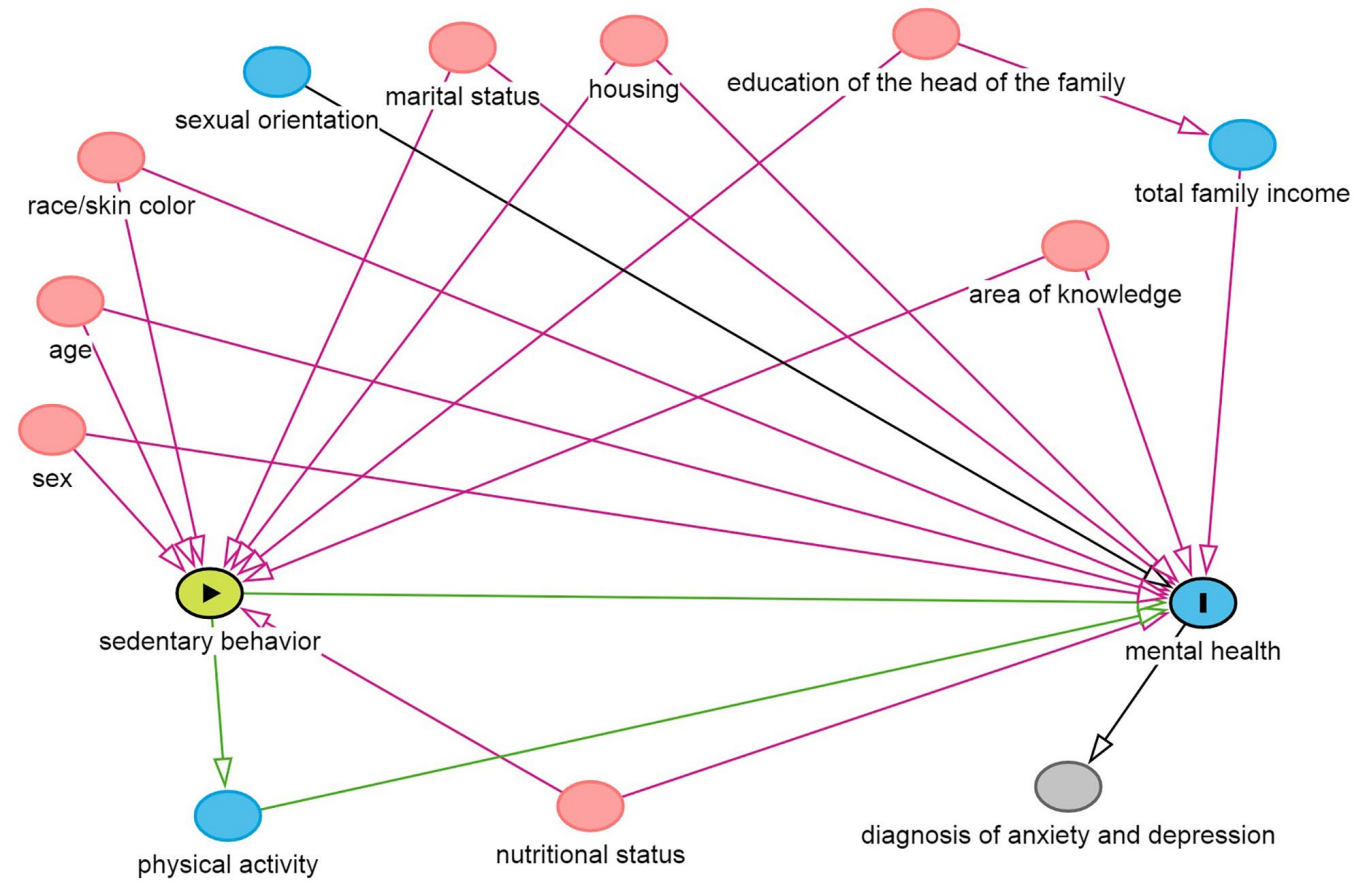
Directed Acyclic Graph (DAG) of the association between sedentary behavior and mental health Legend: DAG: Directed acyclic graph. The variable in green and with the “►” symbol inside the rectangle was the explanatory variable; in blue and with the letter “I” inside is the outcome variable, composed of anxiety symptoms and depression symptoms. The figure shows only the variables that were selected for multivariable. The adjustment variables are: biological sex, age, skin color, marital status, housing, education of the head of the family, area of knowledge and excess weight. The arrows indicate the causal relationships between the variables.

A theoretical causality model using a Directed Acyclic Graph (DAG) was created to select the appropriate adjustment variables, considering the outcome (symptoms of anxiety and depression) and exposure (SB). To elaborate the DAG, the online software Dagitty version 3.2 was used. To avoid unnecessary adjustments, spurious associations and estimation errors, a minimum set of variables was defined for the analyses, according to the back door criterion. The multivariable model was adjusted for biological sex, age, skin color, marital status, housing, education of the head of the family, area of knowledge and excess weight (Fig. 1).

To verify the association between the explanatory variables and the outcome, crude and adjusted logistic regression was performed, with Odds ratio (OR) and 95% confidence interval (95% CI). In the multivariable logistic models, it was decided to include only the variable of education of the head of the family, without adjusting for the students’ family income. The choice is justified by considering that the variables are collinear and that, in the academic environment, the parental education may be more important in students’ persistence at university, reducing dropout rates.

The level of statistical significance adopted for all analyzes was 5%. Analyses were performed using Stata version 13.0 (Stata Corporation, College Station, TX, USA).

## Results

Among the 8,650 students evaluated, the majority were female (65.7%), self-declared white (55.4%), heterosexual (68.2%), single (90.6%) and living with family members (76 0.3%). The average age of the participants was 23.9 ± 6.34 years. Regarding the students’ family cycle, 39.4% were children of parents with completed higher education and 40.9% reported a family income of 3 to 5 minimum wages. Students mentioned being enrolled in the following areas of knowledge: 39.5% exact sciences, 31.6% in life sciences and 28.9% of human, social and applied sciences. When evaluating movement behaviors, it was observed that 55.1% of students were classified with high SB (≥ 9 h/day) and 52.4% engaged in less than 2.5 min of MVPA per sedentary hour. Approximately 35.0% of students were classified as overweight. The main characteristics of the participants are presented in Table 1.

**Table 1:** Sociodemographic characteristics, academic aspect and health condition according to mental health, PADu-multicenter study (2021/2022)

Variables	Total (*n*,%)	Symptoms of anxiety		*P* value^a^	Symptoms of depression		*P* value^a^
		Normal and mild (*n* = 3.486, 40.3%)	Moderate to extremely severe (*n* = 5.164, 59.7%)		Normal and mild (*n* = 3.201, 37.0%)	Moderate to extremely severe (*n* = 5.449, 63.0%)	
**Biological sex** (***n*** = 8.615) ^a^				< 0.001			< 0.001
Male	2.955 (34.3)	1.560 (52.8)	1.395 (47.2)		1.234 (41.8)	1.721 (58.2)	
Female	5.660 (65.7)	1.913 (33.8)	3.747 (66.2)		1.959 (34.6)	3.701 (66.4)	
**Age**				**< 0.001**			**0.006**
18 to 20 years	2.552 (29.5)	969 (38.0)	1.583 (62.0)		977 (38.3)	1.575 (61.7)	
21 to 22 years	2.135 (24.7)	855 (40.0)	1.280 (60.0)		789 (37.0)	1.346 (63.0)	
23 to 25 years	2.000 (23.1)	759 (38.0)	1.241 (62.0)		677 (33.9)	1.323 (66.1)	
≥ 26 years	1.963 (22.7)	903 (46.0)	1.060 (54.0)		758 (38.6)	1.205 (61.4)	
**Race/skin color (** *n* ** = 8.474)** ^**a**^				**0.016**			**< 0.001**
White	4.694 (55.4)	1.953 (41.6)	2.741 (58.4)		1.833 (39.0)	2.861 (61.0)	
Brown	2.622 (30.9)	1.047 (39.9)	1.575 (60.1)		961 (36.7)	1.661 (63.3)	
Black	1.039 (12.3)	382 (36.8)	657 (63.2)		307 (29.6)	732 (70.4)	
Yellow/indigenous/other	119 (1.4)	55 (46.2)	64 (53.8)		45 (37.8)	74 (62.2)	
**Sexual orientation (** ***n*** ** = 8.374)** ^**a**^				**< 0.001**			**< 0.001**
Heterosexual	5.714 (68.2)	2.614 (45.8)	3.100 (54.2)		2.412 (42.2)	3.302 (57.8)	
Homosexual	753 (9.0)	254 (33.7)	499 (66.3)		217 (28.8)	536 (71.2)	
Bisexual	1.685 (20.1)	467 (27.7)	1.218 (72.3)		448 (26.6)	1.237 (73.4)	
Asexual and other	222 (2.7)	51 (23.0)	171 (77.0)		37 (16.7)	185 (83.3)	
**Marital status (** ***n*** ** = 8.582)** ^**a**^				**< 0.001**			**< 0.001**
Sigle	7.775 (90.6)	3.067 (39.4)	4.708 (60.6)		2.819 (36.3)	4.956 (63.7)	
Married/stable union	709 (8.3)	356 (50.2)	353 (49.8)		321 (45.3)	388 (54.7)	
Widowed/divorced	98 (1.1)	42 (42.9)	56 (57.1)		44 (44.9)	54 (55.1)	
**Housing**				**0.061**			**0.211**
With family members	2.048 (23.7)	2.697 (40.9)	3.905 (59.1)		2.467 (37.4)	4.135 (62.6)	
Without family members	6.602 (76.3)	789 (38.5)	1.259 (61.5)		734 (35.8)	1.314 (64.2)	
**Education of the head of the family (** ***n*** ** = 8.528)** ^**a**^				**< 0.001**			**< 0.001**
No education or incomplete primary education	1.297 (15.2)	429 (33.1)	868 (66.9)		405 (31.2)	892 (68.8)	
Complete primary education or incomplete secondary education	941 (11.0)	371 (39.4)	570 (60.6)		342 (36.3)	599 (63.7)	
Complete secondary education or incomplete higher education	2.931 (34.4)	1.171 (40.0)	1.760 (60.0)		1.035 (35.3)	1.896 (64.7)	
Complete higher education	3.359 (39.4)	1.463 (43.6)	1.896 (56.4)		1.381 (41.1)	1.978 (58.9)	
**Total family income (** *n* ** = 8.090)** ^**a.b**^				**< 0.001**			**< 0.001**
≤ 1 to 2 minimum wages	2.595 (32.1)	812 (31.3)	1.783 (68.7)		754 (29.1)	1.841 (70.9)	
3 to 5 minimum wages	3.310 (40.9)	1.383 (41.8)	1.927 (58.2)		1.219 (36.8)	2.091 (63.2)	
6 to 10 minimum wages	1.397 (17.3)	642 (46.0)	755 (54.0)		603 (43.2)	794 (56.8)	
> 10 minimum wages	788 (9.7)	416 (52.8)	372 (47.2)		404 (51.3)	384 (48.7)	
**Area of knowledge**				**< 0.001**			**< 0.001**
Exact Sciences	3.416 (39.5)	1.494 (43.7)	1.922 (56.3)		1.310 (38.3)	2.106 (61.7)	
Life Sciences	2.731 (31.6)	1.151 (42.1)	1.580 (57.9)		1.092 (40.0)	1.639 (60.0)	
Humanities and Social and Applied Sciences	2.503 (28.9)	841 (33.6)	1.662 (66.4)		799 (31.9)	1.704 (68.1)	
**Sedentary behavior (** ***n*** ** = 8.059)** ^**a.c**^				**< 0.001**			**< 0.001**
< 9 h	3.619 (44.9)	1.614 (44.6)	2.005 (55.4)		1.614 (44.60)	2.005 (55.4)	
≥ 9 h	4.440 (55.1)	1.659 (37.4)	2.781 (62.6)		1.659 (37.4)	2.781 (62.6)	
**Ratio MVPA/SB (8.051)** ^**a. d**^				**< 0.001**			**< 0.001**
≥ 2.5 min of MVPA per hour of SB	3.832 (47.6)	1.752 (45.7)	2.080 (54.3)		1.752 (45.7)	2.080 (54.3)	
< 2.5 min of MVPA per hour of SB	4.219 (52.4)	1.518 (36.0)	2.701 (64.0)		1.518 (36.0)	2.701 (64.0)	
**Nutritional status (** ***n*** ** = 8.609)** ^**a. e**^				**< 0.001**			**< 0.001**
Not overweight	5.604 (65.1)	2.344 (41.8)	3.260 (58.2)		2.195 (39.2)	3.409 (60.8)	
Overweight	3.005 (34.9)	1.130 (37.6)	1.875 (62.4)		994 (33.1)	2.011 (66.9)	

Note:

* Variables with loss of responses. *P* value obtained using bivariate logistic regression; In bold: the statistically significant variables in the bivariate analysis;

^a^ The n of variables with an asterisk (^a^) differ from the n of the study due to loss of response;

^b^ Minimum wage in force in Brazil in 2021 = R$1.100.00 (approximately $223.22);

^c^ Ponto de corte de 9 horas proposto por Ku et al. (2018);

^d^ The MVPA/SB ratio was calculated by dividing the minutes of MVPA per day by the hours of SB per day. Foi adotado o ponto de corte de 2,5 minutos de MVPA por hora de SB proposto por Chastin et al.(2021);

^e^ Nutritional status: not overweight = BMI < 24.9 kg/m^2^; overweight = BMI ≥ 25 kg/m^2^.

Through multivariable regression analysis between movement behaviors and the presence of symptoms of anxiety and depression, it was observed that in classification 1 (< 3 h; 3–6 h; 6–9 h; ≥ 9 h) students with SB of 9 h or more per day presented greater chances symptoms of depression [OR: 1.85 (95%CI: 1.33–2.57)] when compared to students with less than 3 h of SB. No positive association was observed for symptoms of anxiety. In classification 2 (< 9 h and ≥ 9 h), the chance of symptoms of anxiety [OR: 1.37 (95%CI: 1.24–1.50)] and depression [OR: 1.61 (95%CI: 1.47–1.77)] was higher among students with SB of 9 h or more per day compared to those who were classified as having less than 9 h. Students who performed less than 2.5 min of MVPA per hour of SB were more likely to present symptoms of anxiety [OR: 1.44 (95%CI: 1.31–1.58)] and depression [OR: 1.74 (95%CI %: 1.59–1.92)] when compared with students who performed 2.5 min or more of MVPA per hour of SB (Table 2**).**

**Table 2:** Association of movement behaviors with mental health, PADu-multicêntrico study (*n* = 8.059)

Movement behaviors	Presence of symptoms anxiety		*p*-value	Presence of symptoms depression		*p*-value
	Unadjusted analysis	Adjusted analysis*		Unadjusted analysis	Adjusted analysis*	
	OR (CI95%)	OR (CI95%)		OR (CI95%)	OR (CI95%)	
**SB - classification 1**						
< 3 h/day	1	1		1		
3–6 h/day	0.90 (0.64–1.25)	0.89 (0.62–1.27)	0.519	1.00 (0.72–1.40)	1.04 (0.73–1.47)	0.840
6–9 h/day	0.95 (0.69–1.31)	0.92 (0.65–1.30)	0.642	1.17 (0.85–1.61)	1.21 (0.86–1.69)	0.274
≥ 9 h/day	1.26 (0.92–1.73)	1.25 (0.89–1.75)	0.195	1.78 (1.30–2.44)	1.85 (1.33–2.57)	**0.000**
**SB - classification 2**						
< 9 h/day	1	1		1	1	
≥ 9 h/day	1.35 (1.23–1.48)	1.37 (1.24–1.50)	**< 0.000**	1.60 (1.46–1.75)	1.61 (1.47–1.77)	**< 0.000**
**Ratio MVPA/SB**						
≥ 2,5 min of MVPA per hour of SB	1	1		1	1	
< 2,5 min of MVPA per hour of SB	1.50 (1.37–1.64)	1.44 (1.31–1.58)	**< 0.000**	1.80 (1.64–1.97)	1.74 (1.59–1.92)	**< 0.000**

Note: Movement behaviors: Sedentary behavior (SB) and moderate to vigorous leisure-time physical activity (MVPA).

OR: Odds ratio; CI: Confidence interval; min: minutes

The ratio MVPA/SB was calculated by dividing the minutes of MVPA per day by the hours of SB per day. The values presented are the mean and 95% CI of the ratio MVPA/SB for each category of SB.

*Multivariable logistic regression adjusted according to directed acyclic graph. Adjusted for biological sex, age, skin color, marital status, housing, education of the head of the family, area of knowledge and excess weight

Values in bold indicate statistical significance (*p*-value < 0.05)

## Discussion

The results of this study indicate that symptoms of mental disorders and SB were highly prevalent among university students during the COVID-19 pandemic. These findings corroborate the initial hypotheses that university students with higher levels of SB are more likely to experience symptoms of anxiety and depression during the COVID-19 pandemic, as well as those students who do not achieve the level of physical activity recommended by the literature, specifically of 2.5 min or more of MVPA per hour of SB.

In recent years, SB has become a topic of investigation in research due to its high prevalence and growing concern about the potential impact on individuals’ health [22, 30]. Epidemiological studies have demonstrated that increased time spent on SB is associated with adverse health outcomes, including chronic non-communicable diseases (NCDs), mental disorders, and premature mortality [14, 30, 31]. Globally, it is estimated that 3.8% of all-cause mortality in adults is attributed to SB, regardless of physical activity levels [32], making it one of the major public health challenges worldwide [22].

Young college adults, typically aged between 18 and 25 years, have been identified as a population at risk for high levels of SB, given the significant proportion of time spent studying and the nature of the higher education system that incorporates numerous sedentary activities, such as sitting in classrooms or in front of screens for studying [31, 33, 34]. Furthermore, the high prevalence of SB among the university population may be linked to the transitional period that students experience during this phase of their lives, marked by declines in overall well-being [13, 35] and the adoption of independent lifestyles [31].

It is estimated that total SB in young adults varies between 6 and 9 or more hours per day [31, 36, 37]. A cross-sectional study conducted with students in Brazil in 2019, evaluated obesogenic behaviors such as screen time-based SB, observed that 49.2% (95% CI: 44.0–54.5%) of young individuals spend more than 6 h per day in front of screens, such as cell phones, television, computers, or tablets [38]. Considering the scenario of the COVID-19 pandemic and the behavioral changes resulting from implemented strategies, there is evidence indicating that this situation has favored to an increase in SB in the population [15], including university students [6, 34], which may vary according to context and individual characteristics [15].

The inclusion of remote teaching may have contributed to an increase in sedentary time among university students due to several factors, especially increased time spent in front of screens, studying, participating in classes and meetings via video calls and/or following social media [15, 39], and the lack of travel, since, with online classes, students no longer needed to move to the university, which may have reduced, for example, the time spent walking during daily commutes [34].

Along with the high rates of SB, the present study also observed a high prevalence of symptoms of anxiety and depression among participants, corroborating the findings of Valdés and collaborators [40], Barbosa and collaborators [41] and Ke and collaborators [42]. In the case of the university population, the combination of predictive factors related to the COVID-19 pandemic, such as the inclusion of remote teaching, lack of interaction with colleagues, which mainly affected contact and socialization, as well as specific stressors related to academic routine, added to other pre-existing difficulties, introduced new challenges into the routine that placed university students in a more vulnerable position, with possible implications for mental health [42, 43].

In the present study, it was observed that students with SB who did not reach the recommendations of 2.5 min or more of MVPA per hour of SB were more likely to experience symptoms of anxiety and depression during the COVID-19 pandemic. There are some mechanisms that explain the association between high levels of SB and mental disorders.

One of the possible mechanisms is related to prolonged SB, which can cause disturbances in biological pathways. There is evidence to show that SB, especially when associated with excessive screen use (television or cell phone), can lead to loneliness, affect interpersonal relationships, and increase central nervous system arousal, which could potentially elevate anxiety levels and create low emotional stability [14, 44, 45]. Indirect mechanisms linking the association of SB and mental health can be explained by intermediate factors such as obesity, unhealthy eating habits, and sleep disorders [15, 44, 45]. Furthermore, increased time spent on SB may reduce physical activity, thereby limiting the potential benefits of exercise and/or body movement on mental health [44].

Some hypotheses can explain how low levels of physical activity and SB can contribute to the development of mental disorders, with emphasis on biological, psychosocial and/or common cause mechanisms. The practice of physical activity has a beneficial effect on the HPA system, and dysregulation of this HPA axis and hypersecretion of cortisol can adversely affect mental health. In this sense, sufficient physical activity can act as a moderator to reduce cortisol secretion levels, which is a hormone responsible for various responses to stress episodes. In turn, low levels of physical activity can interfere with the functioning of the HPA axis, alter serum cortisol levels and favor the development of mental disorders [16].

Studies show that increasing levels of physical activity and decreasing time spent on SB are important public health objectives, mainly in to reduce the harmful effects of SB on health, whether physical or mental [11, 12]. These findings are in line with the WHO 2020 global physical activity guidelines, which recommend that all adults, aged 18 to 64, should limit the amount of time spent in SB and that replacing sedentary time with physical activity of any intensity brings health benefits, highlighting the important role of physical activity in promoting the health of individuals [10].

Although the findings provide relevant insights, this study presents some limitations that must be considered when interpreting the results. First, the information obtained is self-reported, which may have generated memory bias, leading to overestimation and/or underestimation of the data. The presence of symptoms of anxiety and depression was assessed using a self-reported scale; therefore, it does not provide a medical diagnosis of mental disorders but rather a score that classifies the symptoms. Physical activity, for example, was assessed only in the leisure domain, which may lead to an underestimation of the total time spent practicing physical activity. Even with the possibility of this bias, the findings of the present study are consistent with the literature. Another limitation refers to the methodological design of the study, which, due to its cross-sectional nature, does not allow inferring causality between SB and the presence of symptoms of anxiety and depression. Even after making the necessary adjustments for potential confounders, based on the DAG approach, the presence of residual or unmeasured factors that could affect the association cannot be ruled out. Finally, while we recognize that the 2.5-minute MVPA/SB cutoff proposed by Chastin et al., adopted in our study, may have limitations, we believe that this cutoff provides an important initial basis for analyzing the relationship between physical activity and SB. It can provide evidence for future integrated recommendations that consider the interactions between different movement behaviors, such as physical activity and SB, in the university context. This is particularly relevant for our study population, composed of university students who exhibit high levels of SB.

Considering the design of this study, longitudinal research is needed to confirm our findings and clarify the direction of the association between SB and symptoms of anxiety and depression in the university population. Additionally, it is important to continue studying the relationship between these variables using objective measurement instruments to complement the information provided by self-report questionnaires. For example, an accelerometer could be used to measure SB and physical activity, allowing for a more precise recording of movement behaviors. For future studies, it is suggested that physical activity be assessed in other domains besides leisure time (in occupational activities, commuting, and household activities), which will allow the construction of multiple indicators of physical activity patterns. Furthermore, it is necessary to conduct studies that test cut-off points to determine how much physical activity is required to minimize the negative effects of SB among students. For example, if a student spends 9 h in SB, it is important to identify how much physical activity they need to perform to reduce the negative impacts associated with SB. Understanding this relationship can help develop practical and effective recommendations to promote the mental and physical health of university students.

Despite the aforementioned limitations, the present study has strengths. To our knowledge, this is the first study to evaluate the association of SB with the presence of anxiety and depression symptoms and the relationship between MVPA and SB in university students during the COVID-19 pandemic in Brazil. Furthermore, as another positive point, the epidemiological panorama stands out, especially when considering that this study was conducted during remote teaching with a large sample of university students from different areas of knowledge in Brazilian public universities, a fact that has been little explored in the literature. A differential aspect of our study is that most research evaluates only students from the health field. Our study advances by including students from three areas of knowledge: life sciences, exact sciences, and applied human and social sciences. Furthermore, the findings of the present study allow us to advance the elucidation of how the pandemic may have affected the mental health of the university population, also considering the prevalence of SB.

This study provides important insights into the relationship between time spent in physical activity and SB and mental health outcomes among college students. Our analysis highlights a new method for generating evidence on the health risks/benefits (here for symptoms of anxiety and depression) associated with combinations of time spent in different movement behaviors, including physical activity and SB. By addressing this relationship, we contribute to the understanding of physical activity, SB, and mental health in this specific context. In terms of practical implications, by highlighting the importance of physical activity in promoting mental health, this study can support public policies and interventions aimed at improving the psychological well-being of university students, who are often exposed to prolonged periods of SB due to the characteristics of higher education. Universities should encourage practices that promote a more active lifestyle, such as creating environments that encourage movement, providing exercise facilities within the institution, and establishing rest areas with tables for outdoor studying. Raising awareness among educators about the importance of movement and mental health can contribute to a healthier and more supportive academic environment for university students. These advances are crucial to guide future efforts in the prevention and treatment of mental disorders in this population.

## Conclusion

The findings of this study suggest that SB is an moviment behavior associated with the presence of symptoms of anxiety and depression among university students. Furthermore, the practice of MVPA among individuals with SB is associated with higher chances of symptoms of mental disorders.

These findings reinforce the need for public policies and integrated intervention actions focused on reducing the time spent on SB and improving the practice of physical activity. Universities must promote an opportune and motivating environment for healthy habits, with an emphasis on physical activity and reducing sitting time, to improve mental health and reduce the risk of young adults developing NCDs.

## Data Availability

The datasets generated and/or analyzed as part of the current study are not publicly available due to confidentiality agreements with subjects. However, they can be made available solely for the purpose of review and not for the purpose of publication from the corresponding author upon reasonable request.
